# Mild Hypothermia Attenuates the Anesthetic Isoflurane-Induced Cytotoxicity

**DOI:** 10.3389/fncel.2017.00015

**Published:** 2017-02-08

**Authors:** Cheng Li, Yuanlin Dong, Dan Chen, Zhongcong Xie, Yiying Zhang

**Affiliations:** ^1^Shanghai Tenth People's Hospital, Tongji University School of MedicineShanghai, China; ^2^Geriatric Anesthesia Research Unit, Department of Anesthesia, Critical Care and Pain Medicine, Massachusetts General Hospital and Harvard Medical SchoolCharlestown, MA, USA

**Keywords:** anesthesia, DNA damage, hypothermia, isoflurane, cytotoxicity

## Abstract

The commonly used inhalation anesthetic isoflurane has been reported to induce DNA damage and cytotoxicity. However, the methods to attenuate these effects remain largely to be determined. Mild hypothermia has neuroprotective effects. We therefore set out to assess whether mild hypothermia could protect the isoflurane-induced DNA damage and cytotoxicity. Moreover, we investigated the underlying mechanisms by assessing the effects of mild hypothermia on the isoflurane-induced changes in ATP levels. H4 human neuroglioma cells were treated with 2% isoflurane for 3 or 6 h with and without mild hypothermia (35°C). We assessed the cell viability by using 3-(4,5-Dimethylthiazol-2-yl)-2,5-Diphenyltetrazolium Bromide (MTT) and lactate dehydrogenase (LDH) assay. We determined DNA damage by measuring levels of phosphorylation of the histone protein H2A variant X at Ser139 (γH2A.X), the marker of DNA damage. We also measured ATP levels in the cells. Here we showed that the treatment with 2% isoflurane for 6 h induced cytotoxicity and DNA damage in the cells. Moreover, the treatment with 2% isoflurane for 3 h decreased ATP levels without inducing cytotoxicity. Mild hypothermia attenuated the isoflurane-induced cytotoxicity, DNA damage, and ATP reduction in the cells. Taken together, these data suggest that the isoflurane-induced reduction in ATP levels occurred before the isoflurane-induced cytotoxicity. Isoflurane may induce DNA damage and cause cytotoxicity through reducing ATP levels. Mild hypothermia would ameliorate isoflurane-induced DNA damage and cytotoxicity by attenuating the isoflurane-induced reduction in ATP levels. These pilot studies have established a system and will promote the future investigations of anesthesia neurotoxicity.

## Introduction

Inhalation anesthetic isoflurane has been reported to induce the neurotoxicity associated with Alzheimer's disease (AD) neuropathogenesis [Eckenhoff et al., 2004; Loop et al., 2005; Wei et al., 2005, 2007; Xie et al., 2006, 2007; Lin and Zuo, 2011, reviewed in Vutskits and Xie (2016)]. Moreover, our recent studies have shown that isoflurane can also cause DNA damage (Ni et al., 2016). Hypothermia has been classified into: mild (34.5–36.5°C), moderate (34.5–32°C), marked (28–32°C), and profound hypothermia (<28°C) (Tisherman et al., 1999; Nagel et al., 2008). Hypothermia has been shown to decrease excitotoxicity, inflammation, free radical levels, and intracellular calcium overload, which therefore would protect the apoptosis (Andresen et al., 2015). Mild hypothermia (e.g., 35°C) is a neuroprotective strategy and could be useful to treat brain injuries (Andresen et al., 2015). However, it remains unknown whether mild hypothermia can protect the isoflurane-induced DNA damage and cytotoxicity.

During the cellular senescence or cell death, DNA damage decreases cellular replication, and contributes to the onset of the aging process (Hoeijmakers, 2009; López-Otín et al., 2013). As an aging maker, DNA damage is also associated with neurodegenerative disease (Coppedè and Migliore, 2015; Shiwaku and Okazawa, 2015), brain tumors (McKinnon, 2009), and AD (McKinnon, 2009; Canugovi et al., 2012). Histone protein H2A variant X at Ser139 (γH2A.X) has been reported as one of the markers for DNA damage (Bonner et al., 2008; Garcia-Canton et al., 2012; Valdiglesias et al., 2013; Ni et al., 2016), and isoflurane has been shown to elevate the levels of γH2A.X in H4 human neuroglioma cells (Ni et al., 2016). Therefore, we assessed the interaction of isoflurane and mild hypothermia on the levels of γH2A.X in the cells.

Moreover, hypothermia can decrease the metabolic rate, which may then prevent the reduction in ATP levels (Andresen et al., 2015). Isoflurane has been shown to decrease ATP levels *in vitro* (Zhang et al., 2012). We therefore assessed the interaction of mild hypothermia and isoflurane on the DNA damage, cytotoxicity, and ATP levels in cultured cells. The objective of the current study was to establish a system of interaction of mild hypothermia and isoflurane in cells and prove a concept that mild hypothermia might protect the isoflurane-induced DNA damage and cytotoxicity. The hypothesis in the present study was that mild hypothermia attenuated the isoflurane-induced DNA damage and cytotoxicity though preventing the isoflurane-induced reduction in ATP levels.

## Materials and methods

### Cell line

H4 human neuroglioma cells (H4 cells) were used in the studies. The cells were cultured in DMEM (high glucose) containing 9% heat-inactivated fetal calf serum, 100 U/ml penicillin, 100 ug/ml streptomycin, and 2 mM L-glutamine.

### Cell treatments

Isoflurane was delivered from an anesthesia machine to a sealed plastic box in a 37°C incubator containing 6-well plates; the 6-well plates were seeded with one million cells in 1.5 ml cell culture media per well, as described in our previous studies (Zhang et al., 2012). A Dash 400 gas analyzer (General Electric Company, Boston, MA) was used to continuously monitor the delivered concentrations of carbon dioxide, oxygen, and isoflurane. The cells were treated with 2% isoflurane, plus 21% O_2_, and 5% CO_2_ at for a duration of 3 or 6 h as described by our previous studies (Xie et al., 2006, 2007; Ni et al., 2016). Mild hypothermia (35°C) was created by putting the sealed plastic box in a 35°C incubator.

### Cell viability study

Cell viability was determined by using 3-(4,5-dimethylthiazol-2-yl)-2,5-diphenyl tetrazolium bromide (MTT) (Sigma, St. Louis, MO). Experiments were performed as described in the protocol provided by the company. Briefly, we added 150 μL of MTT (5 mg/mL) solution to each well, containing 1.5 mL of cell culture medium, on a 6-well plate for treatment with 2% isoflurane for 6 h. We then returned the cells to the incubator for 2 h. Finally, we removed the cell culture medium and added 1.5 mL isopropanol into each well. We spectrophotometrically measured the absorbance at a wavelength of 570 nm. We presented the changes in absorbance, as the levels of cell viability, in the cells treated with isoflurane and/or mild hypothermia as the percentage of those in the cells treated with control conditions. The reduction in cell viability (e.g., decrease in MTT levels) indicates cytotoxicity.

### Lactate dehydrogenase release (LDH)

Cell membrane integrity or cell viability was also assessed using a commercial lactate dehydrogenase (LDH) kit (Roche Applied Science, Madison, WI). Experiments were performed as described in the protocol provided by the company. Levels of LDH released into the medium were used to reflect cell membrane integrity or cell viability. Briefly, after the treatments of isoflurane and/or mild hypothermia, we collected the cell culture medium and assessed LDH levels according to the manufacturer's instructions. We spectrophotometrically measured the absorbance at a wavelength of 490 nm. The ratio of LDH levels in the supernatant to the total LDH levels served as the index of cell viability. We presented the changes in absorbance, as the degree of cell viability, in the cells treated with isoflurane and/or mild hypothermia as the percentage of those in the cells treated with control condition. The reduction in cell membrane integrity (e.g., increase in LDH levels) or cell viability indicates cytotoxicity.

### Cell harvest, cell lysis, and protein quantification

The cells were harvested at the end of the experiments for Western blot analysis. The pellets of the harvested cells were detergent-extracted on ice using an immunoprecipitation buffer plus protease inhibitors, as described in our previous studies (Zhang et al., 2012). The lysates were collected, centrifuged at 13,000 rpm for 15 min, and quantified for total protein amount by a bicinchoninic acid protein assay kit (Pierce, Rockford, IL).

### Western blot analyses

Western blot analyses were performed as described in our previous studies (Zhang et al., 2010, 2012). Specifically, γH2A.X antibody (1:1000 dilution; Cell Signaling Technology, Danvers, MA) was used to detect the levels of γH2A.X (15 kDa), and β-actin antibody (1:10,000, Sigma, St. Louis, MO) was used to detect β-actin (42 kDa). Each band in the Western blot represented an independent experiment. The results were averaged from six independent experiments. The intensity of signals was analyzed using the National Institute of Health image program. We quantified the Western blots in two steps. First, we used β-actin levels to normalize protein levels (e.g., determining the ratio of γH2A.X to β-actin amount) and to control for loading differences in the total protein amount. Secondly, we presented protein level changes in cells exposed to isoflurane and/or mild hypothermia as the percentage of those in the control group. One hundred percentage of the protein level changes refer to the control levels, for the purpose of comparison to the experimental conditions.

### ATP measurement

We employed the ATP Determination Kit (Invitrogen) in the experiments to detect ATP levels, as described in our previous studies (Zhang et al., 2010, 2012). In short, the cells were placed in 6-well plates in the incubator overnight. The cells were then exposed to the isoflurane and/or mild hypothermia treatment. At the end of the treatment, the amount of fluorescence was measured, and the levels of ATP in the experimental samples were calculated from the standard curve made from samples containing known amounts of ATP.

### Statistical analyses

Data were expressed as means ± standard deviation (SD). The number of samples was 6–8 per group, and the power calculation was performed using information collected from a preliminary study that was conducted under the same conditions. Based on the preliminary data, assuming a two-sided Student-*t*-test, samples of 6 for each control and treatment group would lead to 90% power and 95% significance. Student's *t*-test and one-way ANOVA were used to determine the difference between the treatment (isoflurane vs. isoflurane plus hypothermia) and control condition. *Post hoc* analyses (Bonferroni test) were conducted if the main effects were found to be statistically significant. *P*-values <0.05 were considered statistically significant. Prism 6 software (GraphPad software, La Jolla, CA) was used to analyze the data.

## Results

### Anesthetic isoflurane induced cytotoxicity in H4 cells

**Figure 1:**
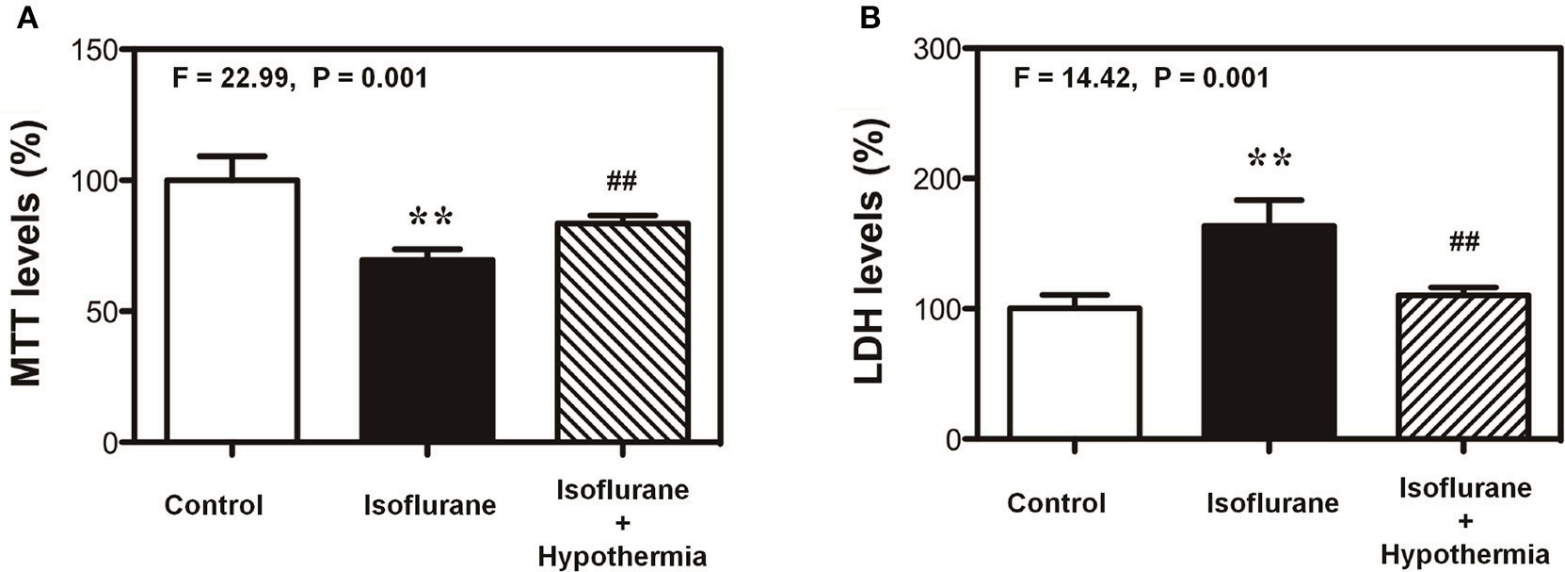
**Mild hypothermia attenuates the isoflurane-induced cells death in H4 cells. (A)** Treatment with 2% isoflurane for 6 h (black bar) decreases the MTT level as compared to the control condition (white bar). Treatment with 2% isoflurane for 6 h plus hypothermia (net bar) leads to lesser reduction in MTT level as compared to isoflurane alone (black bar) in the H4 cells. **(B)** Treatment with 2% isoflurane for 6 h (black bar) increases the LDH level as compared to the control condition (white bar). Treatment with 2% isoflurane for 6 h plus hypothermia (net bar) leads to lesser increase in LDH level as compared to isoflurane alone (black bar) in the H4 cells. 3-(4,5-Dimethylthiazol-2-yl)-2,5-Diphenyltetrazolium Bromide (MTT); lactate dehydrogenase (LDH). *N* = 6–8 in each group. ^**^Indicates that there are significant differences between control condition and isoflurane treatment in MTT levels (Panel **A**) and LDH levels (Panel **B**). ^##^Indicates that there are significant differences between isoflurane treatment and isoflurane plus mild hypothermia treatment in MTT levels (Panel **A**) and LDH levels (Panel **B**).

The objective of the current study was to find the potential methods to protect the cytotoxicity induced by anesthetic isoflurane and to illustrate the underlying mechanisms. Our previous studies had shown that treatment with 2% isoflurane for 6 h could induce cytotoxicity *in vitro* (Xie et al., 2006, 2007; Zhang et al., 2010, 2012). Therefore, we first assessed the effects of mild hypothermia on the isoflurane-induced cytotoxicity (e.g., cell death). The cell viability was determined by MTT assay. As can be seen in Figure 1A, MTT assay showed that treatment with 2% isoflurane for 6 h decreased the MTT levels as compared to control condition: 69.5 vs. 100%. However, mild hypothermia attenuated the isoflurane-induced reduction in MTT levels: 83.5 vs. 69.5%. One-way ANOVA showed the significant difference among these three conditions: control, isoflurane, and isoflurane plus mild hypothermia (Figure 1A, *F* = 22.99, ^**^P = 0.001). Consistently, the mild hypothermia ameliorated the isoflurane-induced increase in LDH release: 100% (control condition) vs. 163 (isoflurane) vs. 110% (isoflurane plus mild hypothermia; Figure 1B, *F* = 14.42, ^**^P = 0.001). These data suggest that isoflurane was able to induce the cytotoxicity in H4 cells, and mild hypothermia could attenuate the isoflurane-induced cytotoxicity in the cells.

### Hypothermia attenuated the isoflurane-induced increase in γH2A.X levels

**Figure 2:**
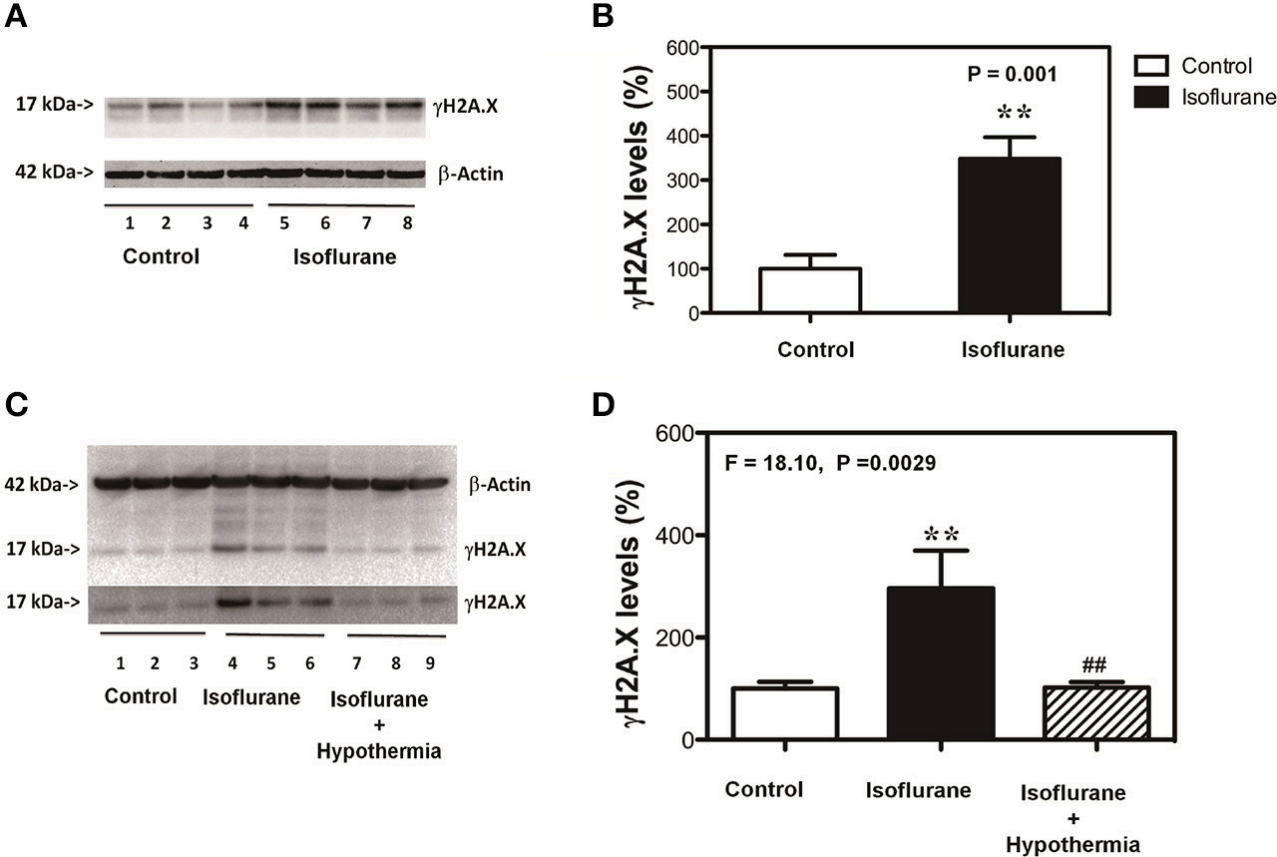
**Mild hypothermia attenuates the isoflurane-induced DNA damage in H4 cells. (A)** Treatment with 2% isoflurane for 6 h (lanes 5–8) increases the γH2A.X level as compared to the control condition (lanes 1–4) in the H4 cells. There is no significant difference in the β-actin level between the isoflurane treatment and control condition. **(B)** Quantification of the Western blot, normalized to β-actin level, shows that the isoflurane treatment (black bar) increases the γH2A.X level as compared to the control condition (white bar) in the cells. **(C)** Treatment with 2% isoflurane for 6 h plus hypothermia (lanes 7–9) leads to lesser increase in the γH2A.X level as compared to the isoflurane treatment alone (lanes 4–6) in the H4 cells. There is no significant difference in the β-actin levels among the isoflurane plus hypothermia, isoflurane treatment alone and control condition. **(D)** Quantification of the Western blot, normalized to β-actin level, shows that the isoflurane treatment (black bar) increases γH2A.X level as compared to control condition (white bar) in the cells. The mild hypothermia treatment (net bar) attenuates the isoflurane-induced increases γH2A.X level (black bar). Phosphorylation of the histone protein H2A variant X at Ser139 (γH2A.X). *N* = 6–8 in each group. ^**^Indicates that there are significant differences between control condition and isoflurane treatment in γH2A.X levels (Panels **B,D**). ^##^Indicates that there are significant differences between isoflurane treatment and isoflurane plus mild hypothermia treatment in γH2A.X levels (Panel **D**).

Given the findings that mild hypothermia was able to attenuate the isoflurane-induced cytotoxicity, next we assessed whether the mild hypothermia could also attenuate the isoflurane-induced increase in the γH2A.X level, the marker of DNA damage, in the cells. The immunoblotting of γH2A.X showed that the treatment with 2% isoflurane for 6 h (lanes 5–8) induced visible increases in the density of bands representing γH2A.X as compared to control condition (lanes 1–4) (Figure 2A). There was no significant difference in β-actin level between the isoflurane treatment and control condition (Figure 2A). The quantification of the Western blot (Figure 2B), based on the ratio of γH2A.X to β-actin, showed that the isoflurane treatment (black bar) increased γH2A.X level as compared to the control condition (white bar): 348 vs. 100%, ^**^P = 0.001. Next, we found that the mild hypothermia (lanes 7–9) could attenuate the isoflurane-induced increase in the γH2A.X level (lanes 4–6) (Figure 2C). The quantification of Western blot showed there was a significant difference on the levels of γH2A.X among the control condition (100%, white bar), isoflurane treatment (318%, black bar), and hypothermia plus isoflurane (108%, shadow bar; *F* = 18.10, ^**^P = 0.0029; Figure 2D). γH2A.X is a maker of DNA damage (Bonner et al., 2008; Garcia-Canton et al., 2012; Valdiglesias et al., 2013; Ni et al., 2016), thus, these data suggest that hypothermia was able to attenuate the isoflurane-induced DNA damage.

### Hypothermia attenuated the isoflurane-induced decrease in ATP levels

**Figure 3:**
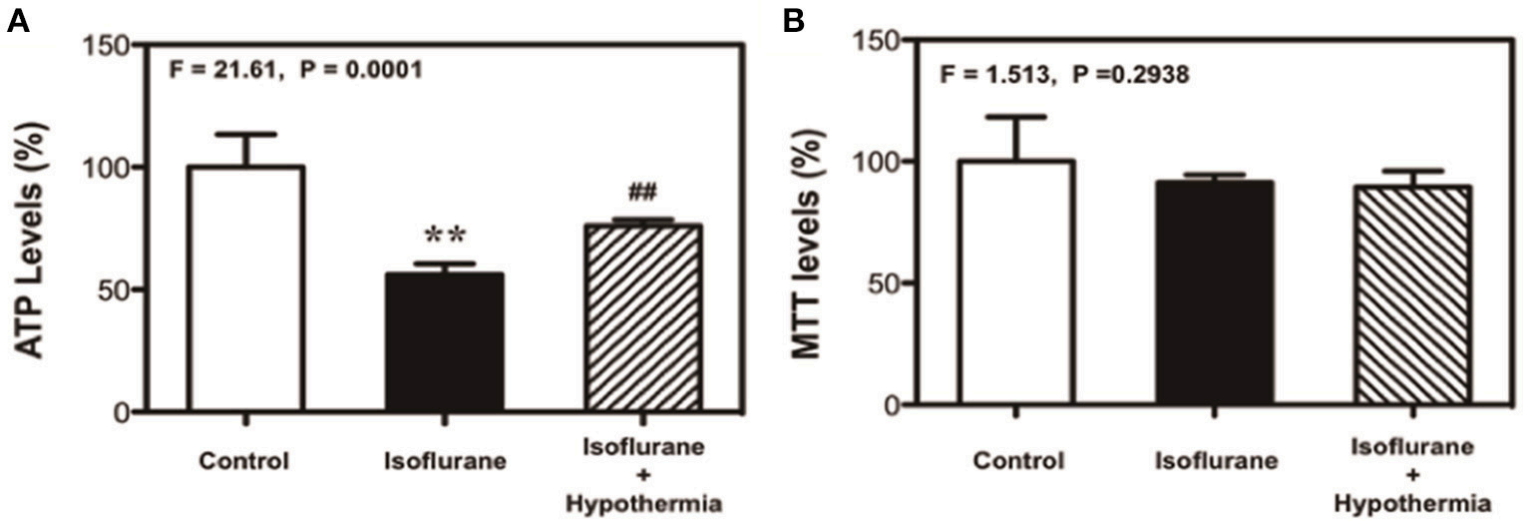
**Mild hypothermia attenuates the isoflurane-induced cytotoxicity though mitigating the ATP reduction in H4 cells. (A)** Treatment with 2% isoflurane for 3 h (black bar) decreases the ATP level as compared to the control condition (white bar). Treatment with 2% isoflurane for 3 h plus hypothermia (net bar) leads to a lesser decreases in ATP level as compared to the isoflurane treatment alone (black bar) in the H4 cells. **(B)** Treatment with 2% isoflurane for 3 h (black bar) or 2% isoflurane for 3 h plus mild hypothermia (net bar) does not significantly affect the MTT levels as compared to the control condition (white bar) in the H4 cells. 3-(4,5-Dimethylthiazol-2-yl)-2,5-Diphenyltetrazolium Bromide (MTT). *N* = 6–8 in each group. ^**^Indicates that there are significant differences between control condition and isoflurane treatment in ATP levels (Panel **A**). ^##^Indicates that there are significant differences between isoflurane treatment and isoflurane plus mild hypothermia treatment in ATP levels (Panel **A**).

Isoflurane has been shown to induce the cytotoxicity by decreasing ATP levels (Zhang et al., 2010, 2012). We therefore determined whether the mild hypothermia could affect the isoflurane-induced changes in ATP levels. In this experiment, we treated the cells with 3% isoflurane for 3 h, but not 6 h to test whether the short time (3 h) isoflurane treatment was able to decrease the ATP levels without causing cytotoxicity in the cells. We found that the treatment with 2% isoflurane for 3 h decreased ATP levels as compared to the control condition, and the mild hypothermia rescued the isoflurane-induced reduction in the ATP levels: control (100%), isoflurane (56%), isoflurane plus mild hypothermia (78%) (Figure 3A, *F* = 21.61, ^**^P = 0.0001, one-way ANOVA). Moreover, we found the treatment with 2% isoflurane for 3 h did not induce cytotoxicity (MTT assay) (Figure 3B). These data showed that isoflurane-induced reduction in ATP levels might occur before the isoflurane-induced cytotoxicity. These results suggest that isoflurane causes DNA damage and induces cytotoxicity by decreasing ATP levels, which can be rescued by the mild hypothermia.

## Discussion

Our previous studies showed that the commonly used anesthetic isoflurane was able to induce neurotoxicity associated with AD neuropathogenesis [(Xie et al., 2006, 2007; Zhang et al., 2010, 2012), reviewed in Vutskits and Xie (2016)] and DNA damage (Ni et al., 2016). Mild hypothermia could protect cytotoxicity (Andresen et al., 2015). In the present studies, we determined whether mild hypothermia (35°C) was able to attenuate the isoflurane-induced cytotoxicity and DNA damage. We found that the mild hypothermia attenuated the isoflurane-induced cytotoxicity (Figure 1) as evidenced by the changes in the levels of MTT and LDH (Figure 1). Then, we were able to show that the mild hypothermia could attenuate the isoflurane-induced DNA damage as evidenced by that the isoflurane-induced elevation in the levels of γH2A.X were reduced by the treatment of hypothermia. Taken together, these data suggest that the mild hypothermia would protect the isoflurane-induced cytotoxicity by mitigating the isoflurane-induced DNA damage. It is unknown whether the mild hypothermia could also attenuate the isoflurane neurotoxicity in mice. The future studies should include the investigation of whether mild hypothermia could rescue the anesthesia-induced neurotoxicity and neurobehavioral deficits in rodents.

Hypothermia could protect the brain and other vital organs during periods of ischemia as well as acute brain injuries (Andresen et al., 2015). Different temperatures may have different effects on cellular functions. Specifically, hypothermia with the temperature of 26–30°C has been reported to induce Tau phosphorylation (Planel et al., 2007, 2009). However, mild or moderate hypothermia could reduce brain damage and neurological deficits before and during cerebral ischemia (Nagel et al., 2008). The future studies should include the systematic investigations to assess the interaction of anesthesia and different temperatures (e.g., 26–35°C) on cell viability, DNA damage and ATP levels.

Mild hypothermia seemed even safer and more effective as compared to moderate hypothermia (WSC, 1997). Thus, it is important to extend the current findings to rodents and even humans in the future to determine whether mild hypothermia could also be used to treat or prevent the postoperative cognitive dysfunction and postoperative delirium in surgical patients.

**Figure 4:**
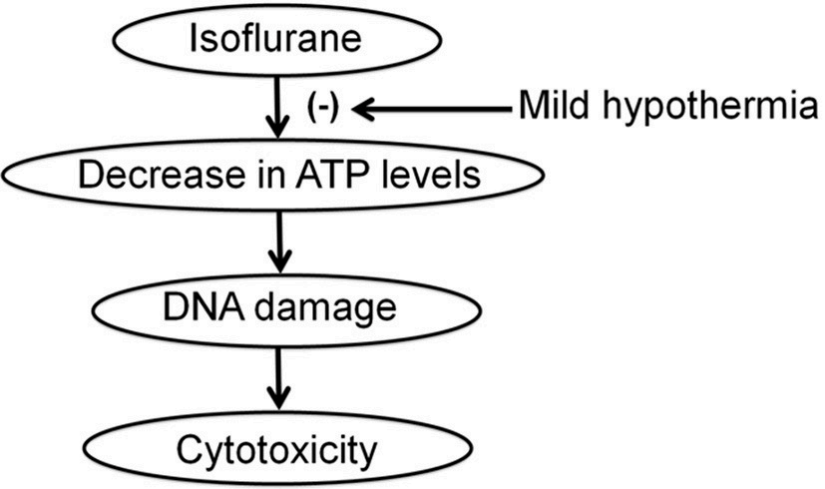
**Hypothesized pathway that mild hypothermia attenuates the isoflurane-induced DNA damage and cytotoxicity**. Isoflurane reduces ATP levels, which induces DNA damage, and consequently cytotoxicity. Mild hypothermia inhibits the isoflurane-induced reduction in ATP levels, which attenuates the isoflurane-induced DNA damage and cytotoxicity.

We further found that mild hypothermia could also rescue the reduction in ATP levels induced by the treatment with 2% isoflurane for 3 h (Figure 3A). Note that this 3 h isoflurane treatment did not cause cytotoxicity (e.g., reduction in MTT levels; Figure 3B). The treatment with 2% isoflurane for 3 h did not cause DNA damage either (Ni et al., 2016). These data suggest that the isoflurane-induced ATP reduction could occur before the isoflurane-induced DNA damage and cytotoxicity in the cells. Collectively, these findings suggest a hypothesized pathway that isoflurane may cause ATP depletion first, which then induces DNA damage and finally cytotoxicity (Figure 4). Moreover, the mild hypothermia could attenuate the isoflurane-induced DNA damage and cytotoxicity by preventing the isoflurane-induced reduction in ATP levels (Figure 4).

The current studies have several limitations. First, we did not compare the effects of different temperatures on the isoflurane-induced cytotoxicity, DNA damage and reduction in ATP levels. However, the major goal of the current studies was to establish a model to assess whether there is an interaction of hypothermia and isoflurane on cytotoxicity, DNA damage and ATP levels. The future studies should include the systematic investigation of the effects of the different temperatures with various times on the isoflurane-induced cytotoxicity, DNA damage and reduction in ATP levels. Second, we did not assess the effects of mild hypothermia (35°C) alone on cell viability, DNA damage and ATP levels. This is mainly because the previous studies have shown that the mild hypothermia (35°C) itself does not significantly affect cell viability and DNA damage in cultured cells (L'Ecuyer et al., 2012). Third, hypothermia has been reported to induce Tau phosphorylation (Planel et al., 2007, 2009). We did not detect the Tau phosphorylation in the current studies. However, the temperature reported to induce Tau phosphorylation was between 26 and 30°C (Planel et al., 2007, 2009). Thus, it was unlikely that the mild hypothermia (35°C) could induce Tau phosphorylation in the cells in the current studies. Moreover, the current studies mainly serve as the proof of a concept that mild hypothermia was able to mitigate the isoflurane-induced cytotoxicity and DNA damage via attenuating the isoflurane-induced reduction in ATP levels. Nevertheless, we will determine the effects of mild hypothermia on Tau phosphorylation in the future studies.

In conclusion, we found that mild hypothermia could attenuate the isoflurane-induced DNA damage, cytotoxicity, and reduction in ATP levels in H4 human neuroglioma cells. Moreover, the inhibition of the reduction in ATP levels could be one of the cellular mechanisms by which mild hypothermia attenuated the isoflurane-induced DNA damage and cytotoxicity. These findings suggest that we could use mild hypothermia to treat or prevent the anesthesia neurotoxicity, pending further investigation.

## Author contributions

CL, YD, and YZ performed experiments, generated and analyzed the data, contributed to project design and manuscript writing. DC and ZX contributed to the design of experiments and data analysis. YZ designed and directed the project, participated in experiments and wrote the manuscript. All authors read and approved the manuscript.

## Funding

This research was supported by R01GM088801, R01AG041274, and R01HD 086977 from National Institutes of Health, Bethesda, Maryland (to ZX). The research was also supported by young investigator research grant (No. 81400879) from National Natural Science Foundation of China (to YZ).

### Conflict of interest statement

The authors declare that the research was conducted in the absence of any commercial or financial relationships that could be construed as a potential conflict of interest. The reviewer JC and handling Editor declared their shared affiliation, and the handling Editor states that the process nevertheless met the standards of a fair and objective review.
